# Might School Performance Grow on Trees? Examining the Link Between “Greenness” and Academic Achievement in Urban, High-Poverty Schools

**DOI:** 10.3389/fpsyg.2018.01669

**Published:** 2018-09-25

**Authors:** Ming Kuo, Matthew H. E. M. Browning, Sonya Sachdeva, Kangjae Lee, Lynne Westphal

**Affiliations:** ^1^Landscape and Human Health Laboratory, Department of Natural Resources and Environmental Sciences, University of Illinois at Urbana-Champaign, Urbana, IL, United States; ^2^Virtual Reality and Nature Lab, Department of Recreation, Sport and Tourism, University of Illinois at Urbana-Champaign, Champaign, IL, United States; ^3^Illinois Informatics Institute, University of Illinois at Urbana-Champaign, Champaign, IL, United States; ^4^United States Department of Agriculture Forest Service, Northern Research Station, Evanston, IL, United States

**Keywords:** geographic information systems, academic performance, greening, schoolyards, socioeconomic status, income, race, urban tree canopy assessment

## Abstract

In the United States, schools serving urban, low-income students are among the lowest-performing academically. Previous research in relatively well-off populations has linked vegetation in schoolyards and surrounding neighborhoods to better school performance even after controlling for important confounding factors, raising the tantalizing possibility that greening might boost academic achievement. This study extended previous cross-sectional research on the “greenness”-academic achievement link to a public school district in which nine out of ten children were eligible for free lunch. In generalized linear mixed models, Light Detection and Ranging (LiDAR)-based measurements of green cover for 318 Chicago public schools predicted statistically significantly better school performance on standardized tests of math, with marginally statistically significant results for reading—even after controlling for disadvantage, an index combining poverty and minority status. Pupil/teacher ratio %bilingual, school size, and %female could not account for the greenness-performance link. Interactions between greenness and Disadvantage suggest that the greenness-academic achievement link is different for student bodies with different levels of disadvantage. To determine what forms of green cover were most strongly tied to academic achievement, tree cover was examined separately from grass and shrub cover; only tree cover predicted school performance. Further analyses examined the unique contributions of “school tree cover” (tree cover for the schoolyard and a 25 m buffer) and “neighborhood tree cover” (tree cover for the remainder of a school’s attendance catchment area). School greenness predicted math achievement when neighborhood greenness was controlled for, but neighborhood greenness did not significantly predict either reading or math achievement when school greenness was taken into account. Future research should assess whether greening schoolyards boost school performance.

## Introduction

In the United States, schools serving predominantly urban, low-income populations are struggling. Sixth graders in the richest school districts are four grade levels ahead of children in the poorest districts; there are large gaps between white children and their black and Hispanic classmates; and the gaps are largest in places with large economic disparities (Reardon et al., 2017). Children who attend urban schools in low-income areas have shown the lowest academic achievement in the country for decades (Bernstein, 1992). In the absence of large-scale, structural solutions to poverty and discrimination, low-cost interventions that help disadvantaged urban children reach their potential are urgently needed.

Recent evidence points to the tantalizing possibility that planting in and around schoolyards could actually boost academic achievement. Three key preconditions for learning—ability to concentrate, manageable levels of stress, and intrinsic motivation to learn—have each been tied to green settings and views. Recent experimental work in a school setting echoes a large body of research on the restorative effects of contact with nature on both attention and stress (for reviews, see Kuo, 2015; Becker et al., 2017); views of greenery from classroom windows improve concentration and reduce both self-reported stress and heart rate, whereas classrooms without green views do not (Li and Sullivan, 2016). Along the same lines, learning in relatively green classrooms, in school gardens, and in natural contexts has been associated with high levels of student interest in numerous studies (e.g., Skinner and Chi, 2012; Alon and Tal, 2015; Lekies et al., 2015; for reviews, see Blair, 2009; Chawla, 2015). And at least one quasi-experimental study has shown teaching course material outdoors boosts students’ intrinsic motivation (Bølling et al., 2018).

Given that concentration and intrinsic motivation to learn are each strong contributors to learning (Rowe and Rowe, 1992; Mantzicopoulos and Morris, 1995; Taylor et al., 2014), and given that stress appears to be an important barrier to learning (Grannis, 1992; Leppink et al., 2016), it seems possible that combining these effects simultaneously within a given student might powerfully aid that student’s ability to learn. These effects might be further compounded in a context in which each student is not only more prepared to learn themselves, but is also surrounded by other students more prepared to learn. If so, we might see systematically better academic performance in children attending greener schools and living in greener surroundings.

Indeed, at least four studies have now tied measures of school and neighborhood greenness to academic performance—even after controlling for important potential confounds (Browning et al., 2018). Matsuoka (2010) found that cafeteria views of trees and shrubs correlated with graduation rates and academic merit awards in high schools across southeastern Michigan. Wu et al. (2014) reported that greenness in 250 m to 2,000 m buffers around Massachusetts public schools predicted standardized test scores. More recently, two studies have tied tree cover to test scores—Kweon et al. (2017) examined the greenness-academic achievement (G-AA) link in public elementary, middle, and high schools in Washington, D.C.; and Hodson and Sander (2017) found the G-AA link in third graders’ reading scores (although not math scores) in St. Paul, MN, United States.

Does the greenness-academic achievement link extend to schools serving predominantly urban, low-income populations? Because previous G-AA research has been conducted in school districts serving relatively few disadvantaged students, it is difficult to say. Minnesota, Michigan, and Massachusetts are each well below the average percentage of students eligible for free and reduced lunch nationwide (48%, U.S. Department of Education, 2017a). The Washington D.C. study did include a substantial proportion of low-income students, with an average of 65% of students eligible for free or reduced lunch. However, nearly one-quarter of public schools serve poorer populations than does the Washington D.C. school district (Rich et al., 2016)—and academic performance drops exponentially with decreases in parental income (U.S. Department of Education, 2017b). Thus it is unclear whether the G-AA link holds in the schools where it may be most needed.

This study examined the G-AA relationship in a predominantly urban, low-income, minority school district in which 90% of students are free lunch eligible and 10% are white. Recent work in this district found no G-AA link, but employed coarse “greenness” measures, did not distinguish between different types of vegetation, and failed to consider potential interactions between green cover and student disadvantage (Browning et al., 2018). The current study addresses each of those limitations and has four aims: to examine the relationship between greenness and academic achievement in the context of disadvantage; to determine the extent to which this relationship is driven by greenness immediately around schools versus in surrounding neighborhoods; to examine the contributions of different kinds of green cover to academic achievement; and to examine the relationship between school greenness and disadvantage.

Our first aim was to examine the relationship between greenness and academic achievement in the context of disadvantage. There are reasons to expect this relationship to hold or even strengthen in low-income urban populations. Previous work in inner-city populations has shown striking benefits of residential greenery on residents, including lower levels of mental fatigue (Faber Taylor et al., 2002), more effective life functioning (Kuo and Sullivan, 2001a), better self-discipline (Faber Taylor et al., 2002), and lower levels of aggression (Kuo and Sullivan, 2001b). Further, the effects of green cover on academic achievement could be stronger in disadvantaged populations—to the extent that violence, crowding, and noise in low-income neighborhoods are likely to result in chronic mental fatigue (Kuo, 1992), the rejuvenating effects of green views and elements might be more needed and larger in children from such neighborhoods.

At the same time, there are reasons to think the greenness–academic performance relationship might be weaker in more disadvantaged schools. The more disadvantaged a school, the more likely it is to restrict or eliminate recess: high-poverty schools are over four times more likely than other schools to forego recess entirely, and schools with predominantly African American student bodies are over 2.5 times more likely to forego recess than predominantly White schools (National Center for Education Statistics [NCES], 2006). Without recess outdoors, students’ experience of any greenery present is limited. As a result, even if a disadvantaged school has an adequate level of green cover, its students might not benefit. Further, there is some indication that disadvantaged schools are less likely to have adequate levels of green cover (Kweon et al., 2017).

In sum, disadvantaged schools might benefit more or less from greening than their relatively well-off counterparts. The current study asks whether the relationship between greenness and academic achievement holds in a predominantly disadvantaged population of schools—and within this population, whether the G-AA relationship is strengthened, weakened, or unaffected in the most disadvantaged schools.

The second aim of this study was to examine the unique contributions of greenness immediately around schools versus in surrounding neighborhoods to academic achievement. In two of the previous G-AA studies, the focus was on large areas extending far beyond the schoolyard: Wu et al. (2014) examined the area around a school within a radius of as much as 1.25 miles, and Hodson and Sander (2017) studied school catchment areas—the area within a school’s attendance boundaries, in which its student body lives. Greenness in these large geographic units can be conceptualized as consisting of two parts—an inner, school zone which corresponds to students’ experience of greenness during the school day, and an outer, neighborhood zone within which students might experience greenness outside of the school day. If students’ experience of greenness during the school day plays a substantial role in academic achievement, that would be good news for school administrators, as that experience seems relatively amenable to intervention. School districts can choose to plant and maintain (or not) trees in their schoolyards with relative autonomy. In an urban landscape, a school’s viewshed is not only a relatively small area but typically consists chiefly of school property and the public rights of way immediately surrounding a school—relatively little private property is involved.

The available evidence suggests that school greenness matters. Two studies have examined the impacts of school greenness on cognitive and academic outcomes. Dadvand et al. (2015) examined cognitive development among 2,600 students in 36 schools and found that children in greener schools showed more rapid cognitive development. School greenness in that study included greenness on school property and within a 50 m buffer around school boundaries; cognitive development was operationalized as children’s gains in working memory and attention over the course of a year. A second study, by Kweon et al. (2017), examined greenness on school property and found that schools with more tree cover performed better on standardized tests even after multiple confounding factors were taken into account.

At a smaller scale than the schoolyard, studies on classroom views of nature suggest the importance of nature in the schoolyard. Matsuoka (2010), Benfield et al. (2015), and Li and Sullivan (2016) studied the effects of classroom views on cognitive and academic outcomes; each showed positive effects. Of these, the Li and Sullivan (2016) findings are of particular note as their use of a randomized controlled experimental design helps build the case for a cause-and-effect relationship between green views and cognitive outcomes in an educational setting. Thus, it is at least plausible that students’ experience of greenness at school—the inner zone in our conceptualization—plays a substantial role in the greenness-academic achievement relationship.

To what extent does students’ experience of greenness in the larger environment (the outer, neighborhood zone in our conceptualization) matter? For neighborhood schools (schools that serve the students in the surrounding neighborhood—as opposed to magnet or charter schools, which serve students district-wide), this larger landscape comprises the bulk of their students’ experience of nature outside of school—at home, through the neighborhood during their commute to and from school, and in the neighborhood after school and on weekends. No studies of which we are aware have directly examined this question. Wu et al. (2014) and Hodson and Sander (2017) found that greenness in the larger landscape *including* the school environment (inner plus outer zone) predicted academic performance, but their studies do not tell us how much of this relationship is driven by school greenness (the inner zone alone), nor how much of it is driven by neighborhood greenness (the outer zone alone), nor how much neighborhood greenness might boost academic performance over and above the effects of school greenness. Dadvand et al. (2015) examined a small part of the larger landscape—the greenness around an individual student’s home and on their commute to school—and found that student home greenness does not predict outcomes and commute greenness only weakly predicts outcomes. Although they did not study the impacts of the neighborhood landscape as a whole, their findings regarding these smaller pieces of the larger landscape suggest that neighborhood greenness plays a relatively unimportant role in cognitive outcomes.

To what extent do neighborhood greenness and school greenness contribute to the relationship between overall greenness and academic achievement? In this study, to determine the relative importance of neighborhood greenness and school greenness, we broke overall greenness into its constituent parts (inner and outer zones) and examined the unique contributions of each, in hopes that the results might help guide future efforts to boost academic achievement through greening. While the findings would necessarily be cross-sectional and not causal, they might suggest where greening-for-academic-achievement efforts offer the highest potential return on investment.

The third aim of this study was to examine the contributions of different kinds of green cover to academic achievement. Previously, Normalized Difference Vegetation Index (NDVI)-based work examined only “greenness” or total vegetative cover over relatively large areas and did not distinguish between different kinds of green cover. However, three studies have used measures other than NDVI. Matsuoka (2010) found that a measure of school cafeteria views of nature in which views incorporating trees and shrubs were designated as more natural than views incorporating only views of grass, positively predicted standardized test scores, graduation rates, and 4-year college plans. Further, in that same study, the percentage of lawn per landscaped area negatively predicted test scores and college plans and did not predict graduation rates. Kweon et al. (2017) found tree cover positively related to school performance in both math and reading test scores; grass/shrub cover was negatively related to achievement in some analyses and not related in others. In Hodson and Sander (2017) study, tree canopy was positively tied to reading performance, and grass/shrub cover was not; neither tree nor grass/shrub cover was tied to math performance. Thus the previous literature would seem to suggest that grass and shrub cover do not contribute to academic achievement whereas tree cover does. In this study, the relationship of tree cover and grass/shrub cover to academic achievement were examined separately in hopes of suggesting what greening-for-academic-achievement efforts might focus on planting for the highest potential return on investment.

This study’s fourth and final aim was to examine the relationship between greenness and disadvantage. Existing research on the relationships between income, race, and access to nature often reflect the general view of trees and parks as pleasant but non-essential amenities. Wealthier areas are, on average, substantially greener than their less well-off counterparts (Zhu and Zhang, 2008), and this difference is so stark that it can be seen from space^1^. In urban settings, both low-income and minority residents have been found to have less access to green cover and green spaces (e.g., Byrne and Wolch, 2009; Landry and Chakraborty, 2009; Wen et al., 2013; Rigolon et al., 2018). And to the extent that the greenness of a neighborhood is likely to be reflected in the greenness of a school situated within it, it is perhaps unsurprising that the racial/ethnic composition of schools is tied to levels of schoolyard green cover, where the percentage of white students predicts a higher level of schoolyard green cover (Kweon et al., 2017).

As green cover is increasingly tied to such important aspects of a healthy, functioning city as residents’ health (Kuo, 2015; South et al., 2015; Browning and Rigolon, 2018), neighborhood crime (Kuo and Sullivan, 2001b; Troy et al., 2012; Kondo et al., 2016) and violence (Kuo and Sullivan, 2001a; Branas et al., 2011; Troy et al., 2012; Wolfe and Mennis, 2012; Kondo et al., 2015), and as green cover has been increasingly tied to academic achievement, the relationship between green cover and disadvantage is of increasing importance. In this study, we examined school and neighborhood green cover in relation to levels of disadvantage in the student bodies served.

In a school district in which nine of ten students, on average, are eligible for free lunch, we examined the relationship between greenness and school-level measures of academic achievement in 318 Chicago public schools. Six potential confounding factors were considered—students’ family income, pupil/teacher ratio, total number of students, students’ race/ethnicity, %bilingual, and %female—and our analyses addressed multicollinearity and spatial autocorrelation.

## Materials and Methods

### Setting and Population

This study examined public elementary schools in Chicago. Chicago Public Schools is the third largest school district in the United States and serves a predominantly low-income minority population. In the 2009–2010 time frame of this study, 87% of third graders were eligible for free lunch, and only 8.7% were White; 45% were African-American, 43% Hispanic, and 3% Asian/Pacific Islander. Twenty-six percent of third graders spoke a language other than English at home and scored “below proficient” on an English language test administered by the Illinois State Board of Education^2^. The Chicago Public Schools are a context in which academic underachievement is of pressing concern: at the time window of this study, almost 60% of its students were not meeting grade standards in reading or math on the Illinois State Board of Education’s Illinois Standardized Assessment Test (ISAT)^3^.

Complete data were available for 395 schools. As a central focus of this study was to compare the contributions of school greenness and neighborhood greenness, we excluded 27 schools without a schoolyard and 10 schools serving students outside their immediate neighborhood. Twenty-three catchment areas were assigned to more than one Chicago Public School; in these cases, we selected the school identified by as primary and excluded the others. An additional 15 schools were removed because they failed multivariate normality criteria in chi-square tests of squared Mahalanobis Distances and visual inspection of Quantile–Quantile plots (Tabachnick and Fidell, 2007). The final sample size was 318 schools.

### Greenness Measures

**FIGURE 1:**
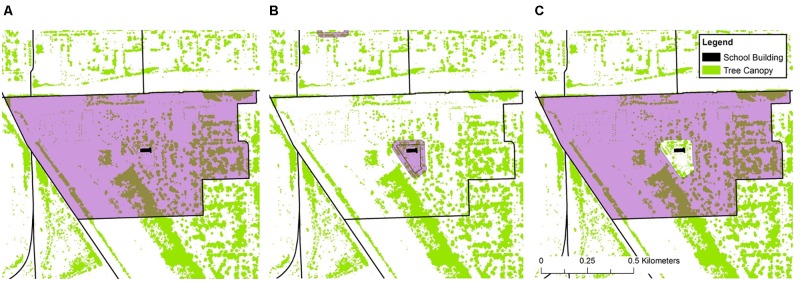
This figure depicts the three geographic areas examined in this study: Catchment **(A)**, School **(B)**, and Neighborhood **(C)**. Catchment comprises all the area within a school’s attendance boundaries and is made up of two non-overlapping components: School, which includes school property and a 25 m buffer around school property, and Neighborhood, which comprises the area inside the attendance areas but outside the school property and 25 m buffer.

Greenness was assessed for each school for two kinds of green cover and three geographic zones. Tree canopy cover and grass/shrub cover were assessed separately; because grass/shrub cover was predominantly composed of grass, we refer to it as grass cover^4^. Greenness for three different geographic zones—catchment, school, and neighborhood—was assessed separately (**Figure 1**). *Catchment* refers to the area a neighborhood school serves, defined by its attendance boundaries; thus catchment greenness refers to the percentage of green cover within the area in which a school’s students live (**Figure 1A**). *Catchment* differs from the radial buffers used in Wu et al. (2014) in that it precisely captures the boundaries of the residential areas of students who attend a given school.

*School* refers to the zone corresponding to students’ experience of nature at school. It encompasses not only any green cover on school property but also in its viewshed as captured in a 25 m buffer around the schoolyard consisting primarily of public rights of way (**Figure 1B**).

*Neighborhood* comprises the area left over when the school area is subtracted from the catchment area—the area inside a school catchment but outside the school zone (**Figure 1C**). This area captures students’ experience of nature on their way to and from school, at home, and in the neighborhood after school and on weekends, other than in the schoolyard or 25 m buffer. It should be noted that *Neighborhood* greenness does not fully represent each student’s out-of-school contact with nature, in that students living near the attendance boundary are especially likely to experience nature outside of the catchment. But it does reasonably approximate the everyday contact with nature that students from a given school are likely to have in common, particularly to the extent that students living near the attendance boundary will experience neighborhood greenness on their way to and from school.

For each of these three geographic zones, each of the two types of greenness was assessed; thus greenness for each school was captured in six variables: *Catchment Trees*, *School Trees*, *Neighborhood Trees*, *Catchment Grass*, *School Grass*, and *Neighborhood Grass*.

Greenness variables were assessed by combining green cover data from the Chicago Urban Tree Canopy Assessment with information about school attendance areas and property boundaries provided by the City of Chicago. The Chicago Urban Tree Canopy Assessment (C-UTC) ^5^, produced by the United States Forest Service and the University of Vermont Spatial Analysis Laboratory, classifies each square meter of land across the City of Chicago into one of seven land cover classifications for the period 2009–2010, including the two used here—tree canopy and grass/shrub—as well as bare earth, water, buildings, roads, and other paved surfaces. These land cover classifications are based on remote sensing data from two sources: Light Detection and Ranging (LiDAR) data and National Agriculture Imagery Program (NAIP) data. LiDAR imagery, collected with a scanning laser instrument mounted onto a low-flying airplane, provided a snapshot of tree and grass/shrub cover (among other kinds of cover) over a 4-day period in April 2009. NAIP, administered by the United States Department of Agriculture, applies object-based image analysis techniques on aerial imagery acquired during the agricultural growing seasons in the United States to extract land cover information (for more information, see MacFaden et al., 2012).

School attendance areas and school property boundaries (based on schools’ tax parcel polygons) were obtained from the City of Chicago. The City of Chicago Data Portal makes this information available for download for free^6^.

By combining C-UTC’s classifications of each square meter of land around schools in our sample with information on school attendance area boundaries and school property boundaries, we calculated the percentage of tree and grass/shrub 1 m^2^ pixels falling within each of our three zones for each of the schools in our sample. All geospatial data processes and buffer creations were performed in ArcMap 10.4.1 (Environmental Systems Research Institute (ESRI), 2016). We calculated the percent canopy cover and percent grass/shrub cover for each polygon by isolating the pixels from the Urban Tree Canopy dataset that fell within, or overlapped with, the polygon of interest using the Tabulate Area tool.

#### Catchment Trees

*Catchment trees* was the percentage of 1 m^2^ pixels falling in or on a school’s attendance boundaries that were classified as tree cover in the Chicago Urban Tree Canopy dataset for 2009–2010. This variable and other greenness variables were centered (recoded, subtracting the average percent tree cover across all schools in our sample) to avoid multicollinearity (see the section “Data Analysis”).

#### School Trees

*School trees* was the percentage of 1 m^2^ pixels falling in or on a school’s property or its viewshed, operationalized as a 25 m buffer around the property classified as tree cover in the same dataset.

#### Neighborhood Trees

*Neighborhood trees* was the percentage of 1 m^2^ pixels falling in or on a school’s attendance boundaries and outside the school property and 25 m buffer classified as tree cover.

#### Catchment Grass

*Catchment grass* was the percentage of 1 m^2^ pixels falling in or on a school’s attendance boundaries classified as grass or shrub cover.

#### School Grass

*School grass* was the percentage of 1 m^2^ pixels falling in or on a school’s property and 25 m buffer classified as grass or shrub cover.

#### Neighborhood Grass

*Neighborhood grass* was operationalized as the percentage of 1 m^2^ pixels falling in or on a school’s attendance boundaries and outside the school property and 25 m buffer classified as grass or shrub cover.

### School Performance and School Characteristics

Information about each school’s performance and characteristics were drawn from the Chicago Public Schools open-source data portal^7^: Reading and math performance on the ISAT, percentage of students eligible for free lunch, percentage of students in different racial/ethnic groups, percentage of female students, total number of students, and pupil/teacher ratio. To align with our geospatial data, data were drawn for academic year 2009–2010.

#### School Performance (Academic Achievement)

School-level academic achievement was operationalized as the percentage of third graders at a school meeting or exceeding expectations in reading and math on the ISAT given in March 2010. While standardized tests have their limitations, they provide a consistent metric for comparing academic achievement across schools, unlike grades, which reflect variation in grading practices from school to school. The ISAT is an assessment developed by the Illinois State Board of Education in coordination with its test development partners. At the time these data were collected, ISAT performance was an important metric at both the student-level and the school-level, playing an important role in decisions of whether a student would be held back a grade, on the one hand (Chicago Public Schools Policy Manual, 2009), and decisions of how much Title I federal funding a school might receive, on the other (van der Klaauw, 2008). Third-grade standardized test performance predicts future outcomes such as high school graduation and college enrollment (Lesnick et al., 2010) and has been used in previous G-AA studies (Wu et al., 2014; Hodson and Sander, 2017).

##### Reading performance

*Reading performance* refers to a school’s performance on the ISAT reading test for school year 2009–2010—specifically, the percentage of third-grade students who met or exceeded the third-grade standard on that test.

##### Math performance

*Math performance* refers to a school’s performance on the ISAT mathematics test for school year 2009–2010—specifically, the percentage of third-grade students who met or exceeded the third-grade standard on that test.

#### Covariates

A number of school characteristics previously found to predict academic achievement were included.

##### Disadvantage

Socioeconomic status and race/ethnicity are each strong predictors of academic achievement. Although neither poverty nor race is destiny, sixth graders in the richest school districts are four grade levels ahead of children in the poorest districts; the average test scores of black students are, on average, roughly two grade levels lower than those of white students in the same district; and the Hispanic-white difference is roughly one-and-a-half grade levels (Reardon et al., 2017). At the school level, income and race/ethnicity are often so strongly associated that including both factors independently and simultaneously in models will risk violating the assumptions of regression due to multicollinearity.

The high correlations between income and race/ethnicity have posed a methodological conundrum for G-AA research which different studies have approached in different ways. Studies by Hodson and Sander (2017) and Kweon et al. (2017) avoided multicollinearity by including only socio-economic status and not race/ethnicity in their regression models. Unfortunately, although income disparities contribute substantially to race differences in academic achievement, race remains a significant source of disadvantage even after income has been taken into account (Bohrnstedt et al., 2015; Reardon et al., 2017). Because race/ethnicity is tied to greenness (Landry and Chakraborty, 2009; Wen et al., 2013; Rigolon et al., 2018) as well as academic achievement (Bohrnstedt et al., 2015; Reardon et al., 2017), leaving race out of greenness-achievement models may entail failing to address a major confounding factor.

Two studies—Matsuoka (2010) and Wu et al. (2014)—included both income and race/ethnicity in their models, thereby addressing both of these potentially important confounds. However, Wu et al. (2014) did not report tests of multicollinearity and subsequent application of their model to a different dataset yielded extremely high levels of multicollinearity, with Variance Inflation Factors in the thousands (Browning et al., 2018)—much higher than even the most liberal suggested threshold of 10.0 (Field, 2014). Matsuoka (2010) reported that multicollinearity was not an issue but did not report Variance Inflation Factor values which would allow readers to assess the extent to which multicollinearity was present.

In this study, we operationalized income as the percentage of students at a school eligible for free lunch and race/ethnicity as the percentage of students identifying as other than white. Income and race/ethnicity were highly correlated (*r* = 0.90, *p* < 0.001), and preliminary analyses showed that including both variables as separate predictors in a model resulted in multicollinearity. To take both income and race/ethnicity into account while avoiding multicollinearity, we combined income and race/ethnicity into a single variable: *Disadvantage*. Combining related but different predictors into a summary index is a statistically robust and theoretically appropriate way to resolve multicollinearity while maintaining the effects of related, but different, concepts (Ahmad et al., 2006). Substituting a combined *Disadvantage* index in place of separate income and race/ethnicity variables reduced all Variance Inflation Factor values to below 3.0, which is the maximum threshold recommended by Zuur et al. (2009) (see **Supplementary Table 1.1**).

*Disadvantage* was the average of two variables: the percentage of students at a school who were eligible for free lunch and the percentage of students at a school not identified as White. *Disadvantage* was centered (recoded, subtracting the mean Disadvantage score) to avoid multicollinearity with the interaction term capturing the interaction between greenness measures and *Disadvantage*.

##### %Bilingual

*%Bilingual* was the percentage of all students in a school whose family spoke a language other than English at home and who scored “below proficient” on an English proficiency language test administered by the Illinois State Board of Education. On average, students who lack English proficiency perform more poorly in school than students who are monolingual (in English) or bilingual but proficient in English (Collier and Thomas, 2004; Han, 2011). Bilingual status has been used as a covariate in two previous G-AA studies: Wu and colleagues (2014) did not report whether it was significantly related to academic achievement; Hodson and Sander (2017) found a significant negative relationship with three of four achievement measures.

##### %Female

%*Female* is the percentage of third graders in a school who were female. These data were not available on the portal and were obtained through a Chicago Public School Office of Accountability Research Review Board External Data Request. Research suggests a gender gap in academic achievement. Historically, most studies demonstrate that boys perform better than girls in mathematics achievement tests (Hyde et al., 1990) – although this gender gap may be subsiding over time (Lindberg et al., 2010). Studies continue to demonstrate girls perform better than boys on reading comprehension tests (Lynn and Mikk, 2009). Only one G-AA study to date, to our knowledge, has included this variable (Wu et al., 2014); although the authors did not report whether %female was related to achievement, they did make two plots of the G-AA relationship, one for schools with more females than average and one for schools with less; because these graphs visually suggested that the G-AA link was stronger for schools with fewer females, we included %female in the covariates examined here.

##### Number of students

*Number of students* is the total number of students at each school. We considered this variable as a potential predictor of academic achievement since the total number of students may influence pupil–teacher ratios (see below) and ultimately the attention and resources given to each student (Mosteller, 1997). While Matsouka (2010) found number of students was non-significant in multivariate models examining greenness and achievement-related outcomes, Kweon et al. (2017) found it was an important covariate in models with greenness and math performance.

##### Pupil/teacher ratio

*Pupil/teacher ratio* is the total number of students divided by the total number of teachers in a school. It is an important indicator of the resources at a school and has been shown to have moderately large effects on test scores and other measures of academic achievement, including in randomized control trials where test scores improve as a direct result of decreasing classroom size (Mosteller, 1997). This variable has been used in two recent G-AA studies, but neither reported a significant relationship with achievement. Wu et al. (2014) provided no results related to pupil/teacher ratio, and Kweon et al. (2017) found no significant relationship with math or reading performance in multivariate analyses; because pupil/teacher ratio was available in our source data and was important to address as a potential confounding variable, we examined it here.

### Data Analysis

Bivariate correlations were used to give an initial picture of which types of greenness (tree cover and grass cover), which components of greenness (school, neighborhood, and catchment) and which potential confounding variables were related to academic achievement.

After conducting bivariate correlations, we tested for spatial autocorrelation. Previous G-AA studies conducted across multiple counties have found spatial autocorrelation (Wu et al., 2014; Hodson and Sander, 2017). The data here were drawn from a single county. To check for within-county spatial autocorrelation, we constructed generalized linear models (GLMs) predicting school performance using *School Trees, Neighborhood Trees,* and *Disadvantage* as predictors and analyzed the residuals from these models for spatial autocorrelation in GeoDa (Anselin et al., 2006). The results showed within-county spatial autocorrelation was present for reading (Global Moran’s *I* = 0.074, *Z* = 2.3, *p* = 0.014). To ensure neither academic achievement model suffered from spatial autocorrelation, we concluded GLM would not suffice for this study.

Chicago was delineated into distinct, stable “Community Areas” in the 1930s by the University of Chicago’s Social Science Research Committee using information from local agencies and the United States Census (Local Community Fact Book Chicago Metropolitan Area, 1990, p. xvii); although the 77 Community Areas were too fine-grained for our purposes (containing in many cases only a single school per area), they are aggregated into nine groups or “sides” which proved to be at an appropriate scale to capture spatial autocorrelation. Generalized linear mixed models (GLMMs) with a random effect for these sides showed no spatial autocorrelation in the residuals (*p* > 0.05).

Accordingly, we used GLMMs with sides modeled as a random effect to examine relationships among green cover, disadvantage, and academic achievement. In this model, we examined the unique contributions of neighborhood and school greenness, respectively, controlled for *Disadvantage*, and included interactions between neighborhood and disadvantage as well as school greenness and disadvantage. For these models, the greenness and disadvantage variables were centered to avoid structural multicollinearity as a consequence of including interactions between greenness and disadvantage.

## Results

**Table 1** provides descriptive statistics. As would be expected from the statistics for the school district as a whole (in which 87% of third graders were eligible for free lunch, and only 8.7% were White), this was, overall, a high-disadvantage sample, with 88% of third graders free lunch eligible and 9.6% White. Given the level of disadvantage, it is perhaps not surprising that school performance was low, with roughly two out every three children not meeting grade-level expectations for reading (63%) and math (66%).

**Table 1:** Descriptive statistics.

Variable (possible range)	Range	Mean ± SD
*Reading performance* (0–100)	6.5–91.1	36.64 ± 17.5
*Math performance* (0–100)	3.4–89.7	34.17 ± 18.42
*Catchment trees*^∗^ (0–100)	0–37.07	12.20 ± 6.99
*School trees*^∗^ (0–100)	2.09–44.70	20.01 ± 7.93
*Neighborhood trees*^∗^ (0–100)	4.33–54.03	19.36 ± 7.43
*Catchment grass/shrub*^∗^ (0–100)	0–56.30	17.17 ± 13.07
*School grass/shrub*^∗^ (0–100)	2.36–52.39	18.25 ± 8.49
*Neighborhood grass/shrub*^∗^ (0–100)	5.26–62.74	22.19 ± 6.67
*%Disadvantaged* (0–100)	19.16–100	89.30 ± 17.97
%Free lunch eligible (0–100)	10.04–100	88.15 ± 18.95
%Non-White (0–100)	20–100	90.44 ± 17.97
%African–American (0–100)	0.1–100	50.41 ± 44.28
%Hispanic (0–100)	0–99.5	37.33 ± 38.27
%Asian (0–100)	0–42	2.48 ± 6.3
%Native American (0–100)	0–2	0.08 ± 0.29
*%Bilingual* (0–100)	0–53	13.34 ± 14.73
*%Female* (0–100)	26–69	49 ± 6
*Number of students* (0–100)	164–2081	643.19 ± 328.51
*Pupil/teacher ratio*	11.73–24.6	18.4 ± 2.16

*N = 318 for all variables. ^∗^Denotes variables which were centered in subsequent GLM analyses to avoid multicollinearity; to calculate their means, minimum values, and maximum values after centering, subtract each by the mean value given in the table.*

**Table 2** shows the bivariate correlations for the variables in this study. As would be expected, schools’ reading and math performance were highly correlated.

**Table 2:** Bivariate correlations among standardized test scores, greenness, and potentially confounding variables, *N* = 318.

	1	2	3	4	5	6	7	8	9	10	11	12	13
1 Reading performance		0.87	0.25	0.37	0.24	0.05	0.07	0.04	−0.74	−0.11	−0.03	−0.02	0.03
2 Math performance	0.87		0.18	0.35	0.18	−0.02	0.05	−0.03	−0.72	0.08	0.13	−0.03	0.09
3 Catchment trees	0.25	0.18		0.41	1.0	0.28	0.14	0.29	−0.34	−0.27	−0.10	−0.01	0.00
4 School trees	0.37	0.35	0.41		0.37	0.02	0.02	0.02	−0.40	0.00	0.10	0.01	0.11
5 Neighborhood trees	0.24	0.18	1.0	0.37		0.29	0.15	0.30	−0.33	−0.28	−0.10	−0.01	0.00
6 Catchment grass	0.05	−0.02	0.28	0.02	0.29		0.35	1.0	−0.05	−0.33	−0.12	−0.08	−0.08
7 School grass	0.07	0.05	0.14	0.02	0.15	0.35		0.31	−0.10	−0.13	−0.04	−0.07	0.00
8 Neighborhood grass	0.04	−0.03	0.29	0.02	0.30	1.0	0.31		−0.04	−0.33	−0.12	−0.07	−0.08
9 %Disadvantaged	−0.74	−0.72	−0.34	−0.40	−0.33	−0.05	−0.10	−0.04		0.06	−0.02	0.02	−0.05
10 %Bilingual	−0.11	0.08	−0.27	0.00	−0.28	−0.33	−0.13	−0.33	0.06		0.58	−0.02	0.23
11 Number of students	−0.03	0.13	−0.10	0.10	−0.10	−0.12	−0.04	−0.12	−0.02	0.58		0.03	0.51
12 %Female	−0.02	−0.03	−0.01	0.01	−0.01	−0.08	−0.07	−0.07	0.02	−0.02	0.03		0.06
13 Pupil/teacher ratio	0.03	0.09	0.00	0.11	0.00	−0.08	0.00	−0.08	−0.05	0.23	0.51	0.06	

*0.095 < |x|≤ 0.113 corresponds to p < 0.1, two-tailed. 0.113 < |x|≤ 0.149 corresponds to p < 0.05, two-tailed. 0.149 < |x|≤ 0.189 corresponds to p < 0.01, two-tailed. 0.189 < |x| corresponds to p < 0.001, two-tailed.*

Tree cover was significantly related to academic achievement. Each of the three tree cover measures (catchment, school, and neighborhood) predicted better reading performance (each with a *p*-value of <0.001), as well as better math performance (*p* < 0.01, *p* < 0.001, *p* < 0.01 respectively), and the Pearson correlation coefficients were of a magnitude that suggested a meaningfully large relationship between tree cover and achievement. Of the three tree cover measures, school trees were more strongly correlated with both reading and math than either neighborhood trees or trees in the catchment as a whole, suggesting that school tree cover might be a more important factor in achievement than neighborhood tree cover.

While all measures of tree cover were significantly tied to academic achievement, the measure including both grass and shrub cover was not related to academic achievement. Neither *Catchment Grass*, nor *School Grass*, nor *Neighborhood Grass* was related to either reading or math performance, indicating that grass (and shrub) cover did not contribute to academic achievement in this study (Aim 3). In subsequent analyses, we focus on the contributions of tree cover to academic achievement and do not examine the contributions of grass or shrub cover further.

Of the various possible confounding variables examined, *Disadvantage* was strongly related to both *Reading Performance* and *Math Performance*. *%Female* and *Pupil/Teacher Ratio* were not related to academic achievement and are not examined further. Also, *Number of Students* was only marginally related to math performance and *%Bilingual* was only marginally related to reading performance. Subsequently, neither of these were considered in subsequent analyses. In summary, we only considered *Disadvantage* in future analyses since other predictors were not statistically significantly related to academic performance.

The bivariate correlations between measures of greenness and disadvantage help address our fourth Aim. Schools serving relatively disadvantaged students were systematically less green in and immediately around their schoolyards: disadvantage was significantly negatively correlated with school tree cover (Pearson correlation coefficient, *r* = −0.40, *p* < 0.001) and marginally significantly negatively correlated with school grass cover (*r* = −0.10, *p* < 0.10). *Disadvantage* was also significantly negatively related to *Neighborhood Trees* and *Catchment Trees* (*r* = −0.33, *p* < 0.001, and *r* = −0.34, *p* < 0.001, respectively), but was not related to neighborhood or catchment grass.

When we examine the means and ranges of tree cover for schools at different levels of disadvantage, the pattern is clear: schools serving more white, well-off students have more tree cover (**Table 3**). In the most disadvantaged quartile, *School Trees* (the percentage of the schoolyard and surrounding 25 m buffer covered by tree canopy) ranged from 0 to 26%, with a mean of 9%; in our least disadvantaged quartile (mean 64% free lunch eligible and 65% non-White), *School Trees* ranged from 0 to 37%, with a mean of l6%). Thus, school tree cover in the extremely disadvantaged schools was roughly half that in less disadvantaged schools (54%).

**Table 3:** The relationship between disadvantage and school tree cover.

Disadvantage quartiles	Range of %school tree cover	Mean of %school tree cover
*Least* (64% free lunch eligible, 65% non-White)	0–37%	16%
*Second* (93%, 97%)	0–31%	12%
*Third* (97%, 99%)	0–26%	11%
*Most Disadvantaged* (99%, 100%)	0–26%	9%

**Table 4** shows results of a GLMM examining the relationships between greenness, disadvantage, and academic achievement while accounting for the part of the county a school belongs to. Accounting for school location was performed by including a random effect variable that identified each school as belonging to one of nine community area groups for the City of Chicago (Local Community Fact Book Chicago Metropolitan Area, 1990). We include the two greenness measures (*School Trees* and *Neighborhood Trees*) but not the third measure linked to academic performance (*Catchment Trees*), because we intended to compare greenness immediately around schools versus greenness in surrounding neighborhoods (Aim 2). Our focus is on greenness due to tree cover but not grass cover, since grass cover did not predict achievement in bivariate correlations. Last, we include the single covariate linked to academic achievement in bivariate correlations (*Disadvantage*) as well as its interaction terms with the two measures of greenness to address Aim 4.

**Table 4:** Using school trees, neighborhood trees, and school disadvantage levels to predict academic achievement in Chicago public schools while accounting for the community area group in which a school is located.

	Math scores		Reading scores	
Predictors	β	*SE*	β	*SE*
School trees	0.22^∗^	0.10	0.18^+^	0.09
Neighborhood trees	−1.59^+^	0.82	−0.45	0.76
%Disadvantaged	−0.78^∗∗∗^	0.05	−0.75^∗∗∗^	0.05
School trees^∗^%Disadvantaged	0.01^∗∗^	0.01	0.01^+^	0.00
Neighborhood trees^∗^%Disadvantaged	−0.02	0.03	0.02	0.03
Marginal R-squared^1^	0.52		0.55	
Conditional R-squared^2^	0.53		0.57	
Moran’s I index	0.030 (*Z* = 0.9), *p* = 0.15		0.029 (*Z* = 1.0), *p* = 0.098	

*^+^<0.10, ^∗^<0.05, ^∗∗^<0.01, ^∗∗∗^<0.001. ^1^R-squared for fixed effects; ^2^R-squared for both fixed and random effects.*

As **Table 4** shows, there was a significant main effect for *School Trees* on math and marginally significant main effect on reading performance, indicating that *School Trees* contribute uniquely to the prediction of academic achievement even after *Neighborhood Trees* are statistically controlled for. *Neighborhood Trees*, however, showed only a marginally significant relationship with math achievement and no relationship to reading achievement once *School Trees* were statistically controlled for. These findings suggest *School Trees* are stronger drivers of academic performance than other types of greenness, including grass cover and trees in surrounding neighborhoods. **Table 4** also shows statistically significant interaction terms between *Disadvantage* and *School Trees* – but not *Neighborhood Trees* – indicating the effects of trees around schools on academic performance vary by levels of disadvantage at the school.

## Discussion

### Summary of Findings

The first aim of this study was to determine whether the G-AA link found in previous studies also held for a highly disadvantaged school district. Previous work in Washington D.C., Minneapolis-St. Paul, and Massachusetts had samples that were composed of 65%, 39%, 35%, and 21% low-income students, respectively (Matsuoka, 2008; Wu et al., 2014; Hodson and Sander, 2017; Kweon et al., 2017). We found that the greenness academic achievement (G-AA) link holds even in a school district where 90% of students were free lunch eligible and fewer than 10% were White. We found a main effect of greenness—more specifically, school tree cover—for school performance in math with a marginally significant main effect for school performance in reading. The advantage of greener schools in math could not be accounted for by levels of disadvantage in their student bodies, nor the percentage of bilingual students, the number of students, the percentage female, nor the pupil/teacher ratio. It appears that the G-AA link holds in schools with high levels of disadvantage.

Interestingly, a significant interaction between school greenness and disadvantage in their relationship to math achievement suggest that the G-AA link is moderated by levels of disadvantage in a student body. Although there was no clear pattern in the relationship, at least three factors may contribute to this moderation. Particularly disadvantaged student bodies may experience chronic mental fatigue, making them more responsive to a greener, more cognitively restorative school environment. On the other hand, among the most disadvantaged schools, the green cover present may not provide a large enough “dose” of green to make a difference in achievement. And finally, a tendency for more disadvantaged schools to forego outdoor recess (National Center for Education Statistics [NCES], 2006) may limit students’ exposure to any green cover present attenuating any G-AA link in these schools.

The second aim of this study was to examine the unique contributions of the greenness immediately around schools and the greenness farther away. In this study, school greenness predicted math achievement and marginally predicted reading achievement even when greenness of the surrounds was taken into account. Neighborhood greenness only marginally predicted math performance when school greenness was considered. This suggests that in previous studies focusing on greenness in the larger landscapes around schools (Wu et al., 2014; Hodson and Sander, 2017), the links between greenness and achievement may have primarily reflected the greenness immediately around the schoolyard and a tendency for greener neighborhoods to also have greener schoolyards. Re-analyses of the data used in those studies (Wu et al., 2014; Hodson and Sander, 2017) in which both near-school and more distant greenness are entered as separate predictors in the same model can tell us whether the previous, positive findings for neighborhood greenness on school performance stand on their own.

The third aim of this study was to examine the contribution of different kinds of green cover. In Chicago Public Schools, we found tree cover to be an important predictor of academic performance, but not grass and shrub cover. This echoes findings in both the Washington D.C. and Minneapolis-St Paul studies, in which trees show significant positive relationships with achievement but grass and shrubs show null or even negative relationships. The current study represents the third city/region in which grass and shrubs have not statistically contributed to academic achievement in public schools. At this time it appears that the link between green cover and achievement is driven primarily by tree cover. Future research should continue to distinguish between tree cover and grass/shrub cover. It is important to note that because measures based on the NDVI do not distinguish between different forms of cover, NDVI-based studies may show no or even negative associations between greenness and academic achievement even if an underlying positive tie between tree cover and achievement exists (Browning et al., 2018).

The fourth and final aim of this study was to examine the relationship between greenness and disadvantage. We found disadvantage was significantly negatively related to greenness, such that the more disadvantaged the student body residing in a neighborhood, the less tree cover existed in the neighborhood and around the school. In schools serving an extremely disadvantaged student body (e.g., 99% free lunch eligible, 100% non-White), tree cover was roughly half (54%) that in schools serving a largely disadvantaged student body (64% free lunch eligible, 65% non-White), and it seems likely that the tree cover in this high-poverty school district falls far short of well-off school districts. Given the research pointing to disease-fighting impacts of contact with nature (Kuo, 2015; South et al., 2015), impacts on crime (Kuo and Sullivan, 2001b; Troy et al., 2012; Kondo et al., 2016) and violence (Kuo and Sullivan, 2001a; Branas et al., 2011; Troy et al., 2012; Wolfe and Mennis, 2012; Kondo et al., 2015) – all of which are critical issues in low-income urban neighborhoods – as well as the possibility that school trees might boost academic achievement, the paucity of tree cover in low-income areas is not merely an aesthetic issue but an important environmental justice issue.

### Methodological Contributions

Three small innovations in this study may be useful in future research. First, in this study, we conceptualized greenness in terms of non-overlapping zones. This separation of zones enabled us to examine the unique contribution of each region to the prediction of academic performance over and above that of other regions. Previous G-AA work has examined entire attendance areas (Hodson and Sander, 2017), schoolyards (Kweon et al., 2017), or increasingly large (overlapping) buffers around schools (Wu et al., 2014), but not mutually exclusive zones; consequently they do not allow the localization of the effects of greenness within larger zones. Second, in our models, we included an interaction term for the moderating effect of disadvantage on the G-AA relationship and centered our disadvantage and greenness variables to prevent multicollinearity. In these data, at least, the interaction was a robust effect and, indeed, without it the relationship between greenness and achievement was consistently significantly *negative* (Browning et al., 2018). And finally, while we are far from the first to combine two conceptually related, highly correlated, uniquely predictive factors into a single index to minimize multicollinearity, the introduction of this practice in to the study of G-AA seems useful and important. Without it, researchers are caught between accepting either extreme instability in estimating effects due to multicollinearity on the one hand and omitting a major confounding variable on the other.

### Limitations

Four characteristics of this study limit the conclusions that can be drawn from its findings. First, because this study was correlational, no conclusions can be drawn regarding whether school greenness is, in fact, affecting school achievement; it is possible that our controls for socioeconomic status and other factors that drive achievement were inadequate. True experiments involving landscape change are typically impossible. However, a new project underway in Louisville, KY, United States may more rigorously address the question of cause-and-effect. Led by The Nature Conservancy and numerous partners including researchers at the University of Louisville, the Green Heart study will rapidly green neighborhoods while other neighborhoods serve as controls, to assess the impacts of greening on a wide range of human health and well-being measures^8^. Second, because we wished to examine the effects of greenery immediately around schools and in the surrounding neighborhood on students living in that neighborhood, we limited our study to neighborhood-based schools. As our sample excludes schools that draw from multiple neighborhoods or even the entire city of Chicago, it is impossible to say whether the G-AA relationship found here extends to magnet, charter, and other multi-neighborhood schools in this city or elsewhere. As these types of schools are growing in number across the United States, assessing the potential contributions of school and residential green on academic achievement for these school types is increasingly important. Third, because we were unable to obtain test scores for individual students, we were able only to study test performance at the level of schools. A study examining test scores and controlling for confounding variables at the individual student level would be stronger. Lastly, like most studies in this line of investigation, this study was cross-sectional, examining only the G-AA relationship in a single school year (2009–2010). However, schools, students, and landscapes change with time. One recent study found that while Chicago Public School test scores were below grade, over time, many Chicago Public School students show significantly more *growth* in test scores than students in other public school systems (Reardon and Hinze-Pifer, 2017). And landscapes can change for the better (i.e., trees planted and maintained, or school yard pavement removed and planted with trees and gardens) or for the worse (i.e., trees lost due to an invasive species like the Emerald Ash borer). A study able to examine test scores *and* changes in greenness over time would complement the existing body of cross-sectional work. A study allowing longitudinal, individual, and school-level modeling of the G-AA relationship would be stronger still.

### Implications for Research and Practice

Given the pressing need to identify feasible ways to boost academic achievement in urban low-income schools, the low cost of greening and its many important ancillary benefits, and the consistent findings in this and previous correlational studies, it is time to conduct field experiments examining standardized test performance in urban, low-income schools randomly assigned to tree planting and control conditions.

Our findings that near-school trees predict performance better than neighborhood trees is good news for school administrators. Schools have jurisdiction over planting and maintenance decisions on their properties; further, much of the 25 m buffers fall on city-owned rights-of-way. Nor would the cost of planting and maintenance necessarily have to fall on the school district. In many cities, there are tree planting programs that purchase and help plant trees on public rights-of-way including school grounds and medians. In Chicago, the non-profit Openlands runs several urban forest-related programs, including “Space to Grow” and “Building School Gardens” that specifically target school landscapes and “TreeKeepers,” which trains volunteers in tree planting and care. Similarly, Philadelphia’s Tree Tenders focuses on schools, and Los Angeles hosts one of the first urban forestry non-profits, TreePeople. Each of these organizations reaches out to schools to assist with landscape transformations and subsequent maintenance, thereby placing meaningful changes in tree cover in reach of even financially strapped school districts.

Greening immediately around schools would cost considerably less than greening the broader neighborhood. Consider the costs entailed in a 10% increase in tree cover in the near-school area versus a school’s attendance area. The average dimension of the schoolyard and its 25 m buffer in our sample was approximately 165 m by 165 m, an area of 27,149 m^2^. The average dimension of the attendance area was approximately 1,228 m by 1,288 m, an area of 1,509,594 m^2^. Assuming that the average installation cost is $100 and the mean crown radius after 10 years of growth is 5 m, each tree would cover 78 m^2^ at the price of $1.28 per square meter. It would take 35 trees to increase tree cover by 10% in the schoolyard + 25 m buffer, but it would take 1,936 trees to increase tree cover by 10% in the surrounding neighborhood. The difference in cost of these two greening efforts is substantial: school greening would cost $3,500, and attendance area greening would cost sixteen times that amount ($193,600) in this scenario.

Another encouraging outcome from our study is that our data further reinforces the importance of trees over grass and shrubs for academic achievement. Increasing tree cover is easier to incorporate into pre-existing landscapes than grasses or shrubs because tree plantings require smaller footprints and create large canopies of “greenness” above. Planting a few trees thus exponentially increases the green cover around schools compared to planting grasses or shrubs, and maintaining growing trees further expands their canopy.

## Conclusion

The study here suggests that greening has the potential to mitigate academic underachievement in high-poverty urban schools. This study also helps guide the *where* and *what* of such efforts. Green cover predicted academic performance even in a highly disadvantaged population of schools. The G-AA link was driven primarily by near-school trees and not by residential tree cover, suggesting that experimental greening efforts might focus on school grounds and the areas within view of the school. Further, tree cover was tied to academic performance, but grass and shrub cover was not, suggesting that experimental greening efforts might focus on planting trees. Finally, even within this high-poverty school district, there was substantial inequity in levels of school tree cover across different levels of disadvantage; we urge researchers and practitioners to conduct field experiments simultaneously addressing this inequity and determining whether its relationship to school performance is causal.

## Author Contributions

MK secured funding, conceptualized the study, guided data analysis, participated in interpretation of data, and lead the write-up. MB prepared data for analysis, guided and conducted data analysis, participated in interpretation of data, wrote substantial portions of the manuscript, and reviewed the manuscript. SS prepared data for analysis, guided and conducted data analysis, prepared tables and figures, and reviewed the manuscript. KL guided selection of GIS and analytic methods, calculated greenness variables, prepared data for analysis, participated in interpretation of data, prepared tables and figures, and reviewed the manuscript. LW provided funding, participated in interpretation of data, and reviewed the manuscript.

## Conflict of Interest Statement

The authors declare that the research was conducted in the absence of any commercial or financial relationships that could be construed as a potential conflict of interest.

## Footnotes

1 http://billmoyers.com/2012/06/10/how-to-spot-income-inequality-from-space-count-the-trees/

2 Please see http://cps.edu/SchoolData/Pages/SchoolData.aspx, specifically in the Demographics section see reports on “Limited English Proficiency, Special Ed, Low Income, IEP” and “Racial/Ethnic” for school year 2009–2010.

3 Please see http://cps.edu/SchoolData/Pages/SchoolData.aspx, especially the Assessment Reports, ISAT reports and Overall reports.

4 http://maps.fieldmuseum.org/CRTI/MuniCanopy/Chicago/Chicago.PDF

5 http://gis.w3.uvm.edu/utc/

6 https://data.cityofchicago.org/

7 http://cps.edu/SchoolData/Pages/SchoolData.aspx

8 https://www.nature.org/newsfeatures/pressreleases/green-heart-project-launches-in-louisville.xml

## References

[B1] Ahmad M. H. Adnan R. Adnan N. (2006). A comparative study on some methods for handling multicollinearity problems. *Matematika* 22 109–119. 10.11113/matematika.v22.n.179

[B2] Alon N. L. Tal T. (2015). Student self-reported learning outcomes of field trips: the pedagogical impact. *Int. J. Sci. Educ.* 37 1279–1298. 10.1080/09500693.1034797

[B3] Anselin L. Syabri I. Kho Y. (2006). *GeoDa*: an introduction to spatial data analysis. *Geogr. Syst.* 35 5–22. 10.1111/j.0016-7363.2005.00671.x

[B4] Becker C. Lauterbach G. Spengler S. Dettweiler U. Mess F. (2017). Effects of Regular Classes in outdoor education settings: a systematic review on students’ learning, social and health dimensions. *Int. J. Environ. Res. Public Health* 14:E485. 10.3390/ijerph14050485 28475167 PMC5451936

[B5] Benfield J. A. Rainbolt G. N. Bell P. A. Donovan G. H. (2015). Classrooms with nature views: evidence of differing student perceptions and behaviors. *Environ. Behav.* 47 140–157. 10.1177/0013916513499583

[B6] Bernstein L. (1992). Where is reform taking place? An analysis of policy changes and school climate. *Educ. Eval. Policy Anal.* 14 297–302.

[B7] Blair D. (2009). The child in the garden: an evaluative review of the benefits of school gardening. *J. Environ. Educ.* 40 15–38. 10.3200/JOEE.40.2.15-38

[B8] Bohrnstedt G. Kitmitto S. Ogut B. Sherman D. Chan D. (2015). *School Composition and the Black–white Achievement Gap (NCES 2015-018).* Washington, DC: National Center for EducationStatistics.

[B9] Bølling M. Otte C. R. Elsborg P. Nielsen G. Bentsen P. (2018). The association between education outside the classroom and students’ school motivation: results from a one-school-year quasi-experiment. *Int. J. Educ. Res.* 89 22–35. 10.1016/j.ijer.2018.03.004

[B10] Branas C. C. Cheney R. A. MacDonald J. M. Tam V. W. Jackson T. D. Ten Have T. R. (2011). A difference-in-differences analysis of health, safety, and greening vacant urban space. *Am. J. Epidemiol.* 174 1–11. 10.1093/aje/kwr273 22079788 PMC3224254

[B11] Browning M. Rigolon A. (2018). Do income, race and ethnicity, and sprawl influence the greenspace-human health link in city-level analyses? Findings from 496 cities in the United States. *Int. J. Environ. Res. Public Health* 15 1–22. 10.3390/ijerph15071541 30037037 PMC6068800

[B12] Browning M. H. E. M. Kuo M. Sachdeva S. Westphal L. Lee K. (2018). Research note: greenness and school-wide test scores are not always positively associated - a replication of “Linking student performance in Massachusetts elementary schools with the ’greenness’ of school surroundings using remote sensing”. *Landsc. Urban Plan.* 178 69–72. 10.1016/j.landurbplan.2018.05.007 25310542

[B13] Byrne J. Wolch J. (2009). Nature, race, and parks: past research and future directions for geographic research. *Prog. Hum. Geogr.* 33 743–765. 10.1177/0309132509103156

[B14] Chawla L. (2015). Benefits of nature contact for children. *J. Plan. Lit.* 30 433–452. 10.1177/0885412215595441

[B15] Chicago Fact Book Consortium (ed.) (1984). *Local Community Fact Book: Chicago Metropolitan Area: Based on the 1970 and 1980 Censuses.* Chicago: Academy Chicago Publishers, Limited

[B16] Chicago Public Schools Policy Manual (2009). *Chicago Public Schools Policy Manual, Elementary School Promotion, adopted.* Available at: http://policy.cps.edu/download.aspx?ID=45 [accessed August 17, 2018].

[B17] Collier V. P. Thomas W. P. (2004). The astounding effectiveness of dual language education for all. *NABE J. Res. Pract.* 2 1–20.

[B18] Dadvand P. Nieuwenhuijsen M. J. Esnaola M. Forns J. Basagaca X. Alvarez-Pedrerol M. (2015). Green spaces and cognitive development in primary schoolchildren. *Proc. Natl. Acad. Sci. U.S.A* 112 7937–7942. 10.1073/pnas.1503402112 26080420 PMC4491800

[B19] Environmental Systems Research Institute (ESRI) (2016). *ArcGIS Release 10.4.1.* Redlands, CA: ESRI.

[B20] Faber Taylor A. Kuo F. E. Sullivan W. C. (2002). Views of nature and self-discipline: evidence from inner city children. *J. Environ. Psychol.* 22 49–63. 10.1006/jevp.2001.0241

[B21] Field A. (2014). *Discovering Statistics using SPSS*, 4 Edn. Thousand Oaks, CA: Sage.

[B22] Grannis J. C. (1992). Students’ stress, distress, and achievement in an urban intermediate school. *J. Early Adolesc.* 12 4–27. 10.1177/0272431692012001001

[B23] Han W.-J. (2011). Bilingualism and academic achievement. *Child Dev.* 83 300–321. 10.1111/j.1467-8624.2011.01686.x 22098584

[B24] Hodson C. B. Sander H. A. (2017). Green urban landscapes and school-level academic performance. *Landsc. Urban Plan.* 160 16–27. 10.1016/j.landurbplan.2016.11.011

[B25] Hyde J. S. Fennema E. Lamon S. J. (1990). Gender differences in mathematics performance: a meta-analysis. *Psychol. Bull.* 107 139–155. 10.1037/0033-2909.107.2.139 2138794

[B26] Kondo M. Hohl B. Han S. Branas C. (2016). Effects of greening and community reuse of vacant lots on crime. *Urban Stud.* 53 3279–3295. 10.1177/0042098015608058 28529389 PMC5436723

[B27] Kondo M. C. Low S. C. Henning J. Branas C. C. (2015). The impact of green stormwater infrastructure installation on surrounding health and safety. *Am. J. Public Health* 105 114–121. 10.2105/AJPH.2014.302314 25602887 PMC4330869

[B28] Kuo F. E. (1992). “Inner cities and chronic mental fatigue: design for a fighting chance,” in *Proceedings of the Environmental Design Research Association*, Oklahoma City, OK.

[B29] Kuo F. E. Sullivan W. C. (2001b). Environment and crime in the inner city: does vegetation reduce crime? *Environ. Behav.* 33 343–367. 10.1177/0013916501333002

[B30] Kuo F. E. Sullivan W. C. (2001a). Aggression and violence in the inner city: effects of environment via mental fatigue. *Environ. Behav.* 33 543–571. 10.1177/00139160121973124

[B31] Kuo M. (2015). How might contact with nature promote human health? Promising mechanisms and a possible central pathway. *Front. Psychol.* 6:1093. 10.3389/fpsyg.2015.01093 26379564 PMC4548093

[B32] Kweon B.-S. Ellis C. D. Lee J. Jacobs K. (2017). The link between school environments and student academic performance. *Urban For. Urban Green.* 23 35–43. 10.1016/j.ufug.2017.02.002

[B33] Landry S. Chakraborty J. (2009). Street trees and equity: evaluating the spatial distribution of an urban Aamenity. *Environ. Plan. A* 41 2651–2670. 10.1068/a41236

[B34] Lekies K. S. Yost G. Rode J. (2015). Urban youth’s experiences of nature: implications for outdoor adventure recreation. *J. Outdoor Recreation Tour.* 9 1–10. 10.1016/j.jort.2015.03.002

[B35] Leppink E. W. Odlaug B. L. Lust K. Christenson G. Grant J. E. (2016). The young and the stressed: stress, impulse control, and health in college students. *J. Nerv. Ment. Dis.* 204 931–938. 10.1097/NMD.0000000000000586 27575792

[B36] Lesnick J. Goerge R. Smithgall C. Gwynne J. (2010). *Reading on Grade Level in Third Grade: How is it Related to High School Performance and College Enrollment*, Vol. 1 Chicago, IL: Chapin Hall at the University ofChicago, 12

[B37] Li D. Sullivan W. C. (2016). Impact of views to school landscapes on recovery from stress and mental fatigue. *Landsc. Urban Plan.* 148 149–158. 10.1016/j.landurbplan.2015.12.015

[B38] Lindberg S. M. Hyde J. S. Petersen J. L. Linn M. C. (2010). New trends in gender and mathematics performance: a meta-analysis. *Psychol. Bull.* 136 1123–1135. 10.1037/a0021276 21038941 PMC3057475

[B39] Lynn R. Mikk J. (2009). Sex differences in reading achievement. *Trames* 13 3–13. 10.3176/tr.2009.1.01

[B40] MacFaden S. W. O’Neil-Dunne J. P. Royar A. R. Lu J. W. Rundle A. G. (2012). High-resolution tree canopy mapping for New York City using LIDAR and object-based image analysis. *J. Appl. Remote Sens.* 6:3567 10.1117/1.JRS.6.063567

[B41] Mantzicopoulos P. Y. Morris D. (1995). A comparison of boys and girls with attention problems: kindergarten through second grade. *Am. J. Orthopsychiatry* 64 522–533. 10.1037/h0079560 7847568

[B42] Markevych I. Schoierer J. Hartig T. Chudnovsky A. Hystad P. Dzhambov A. M. (2017). Exploring pathways linking greenspace to health: theoretical and methodological guidance. *Environ. Res.* 158 301–317. 10.1016/j.envres.2017.06.028 28672128

[B43] Matsuoka R. H. (2008). *High School Landscapes and Student Performance.* Dissertation, University of Michigan, Ann Arbor.

[B44] Matsuoka R. H. (2010). Student performance and high school landscapes: examining the links. *Landsc. Urban Plan.* 97 273–282. 10.1016/j.landurbplan.2010.06.011

[B45] Mosteller F. (1997). The Tennessee study of class size in the early school grades. *Bull. Am. Acad. Arts Sci.* 50 14–25. 10.2307/3824562 8528684

[B46] National Center for Education Statistics [NCES] (2006). *Calories in, Calories out: Food and Exercise in Public Elementary Schools, 2005.* Washington, DC: NCES.

[B47] Reardon S. F. Hinze-Pifer R. (2017). *Test Score Growth Among Public School Students in Chicago Public Schools Policy Manual, 2009-2014.* Available at: http://cepa.stanford.edu/sites/default/files/chicago%20public%20school%20test%20scores%202009-2014.pdf [accessed August 17, 2018].

[B48] Reardon S. F. Kalogrides D. Shores K. (2017). *The Geography of Racial/Ethnic Test Score Gaps. (CEPA Working Paper No. 16-10).* Available at: http://cepa.stanford.edu/content/geography-racialethnic-test-score-gaps [accessed August 17, 2018].

[B49] Rich M. Cox A. Bloch M. (2016). *Money, race and success: how your school district compares. The Upshot: New York Times.* Available at: https://www.nytimes.com/interactive/2016/04/29/upshot/money-race-and-success-how-your-school-district-compares.html

[B50] Rigolon A. Browning M. Jennings V. L. (2018). Inequities in the quality of urban park systems: an environmental justice investigation of cities in the United States. *Landsc. Urban Plan.* 178 156–169. 10.1016/j.landurbplan.2018.05.026

[B51] Rowe K. J. Rowe K. S. (1992). The relationship between inattentiveness in the classroom and reading achievement: part B: an explanatory study. *J. Am. Acad. Child Adolesc. Psychiatry* 31 357–368. 10.1097/00004583-199203000-00026 1564039

[B52] Skinner E. A. Chi U. (2012). Intrinsic motivation and engagement as “active ingredients” in garden-based education: examining models and measures derived from self-determination theory. *J. Environ. Educ.* 43 16–36. 10.1080/00958964.2011.596856

[B53] South E. C. Kondo M. C. Cheney R. A. Branas C. C. (2015). Neighborhood blight, stress, and health: a walking trial of urban greening and ambulatory heart rate. *Am. J. Public Health* 105 909–913. 10.2105/AJPH.2014.302526 25790382 PMC4386540

[B54] Tabachnick B. G. Fidell L. S. (2007). *Using Multivariate Statistics*, 5 Edn. Boston, MA: Allyn & Bacon.

[B55] Taylor G. Jungert T. Mageau G. A. Schattke K. Dedic H. Rosenfield S. (2014). A self-determination theory approach to predicting school achievement over time: the unique role of intrinsic motivation. *Contemp. Educ. Psychol.* 39 342–358. 10.1016/j.cedpsych.2014.08.002

[B56] Troy A. R. Morgan Grove J. O’Neil-Dunne J. (2012). The relationship between tree canopy and crime rates across an urban–rural gradient in the greater Baltimore region. *Landsc. Urban Plan.* 106 262–270. 10.1016/j.landurbplan.2012.03.010

[B57] U.S. Department of Education (2017a). *Number and Percentage of Public School Students Eligible for free or Reduced-price Lunch, by State: Selected years, 2000-01 through 2010-11.* Available at: https://nces.ed.gov/programs/digest/d12/tables/dt12_046.asp [accessed August 17, 2018].

[B58] U.S. Department of Education (2017b). *Promising Results, Continuing Challenges: Final Report of the National Assessment of Title I.* Available at: https://www2.ed.gov/rschstat/eval/disadv/promisingresults/edlite-exsum.html [accessed August 17, 2018].

[B59] van der Klaauw W. (2008). Breaking the link between poverty and low student achievement: an evaluation of Title I. *J. Econom.* 142 731–756. 10.1016/j.jeconom.2007.05.007

[B60] Wen M. Zhang X. Harris C. D. Holt J. B. Croft J. B. (2013). Spatial disparities in the distribution of parks and green spaces in the USA. *Ann. Behav. Med.* 45 18–27. 10.1007/s12160-012-9426-x 23334758 PMC3590901

[B61] Wolfe M. K. Mennis J. (2012). Does vegetation encourage or suppress urban crime? Evidence from Philadelphia, PA. *Landsc. Urban Plan.* 108:112 10.1016/j.landurbplan.2012.08.006

[B62] Wu C.-D. McNeely E. Cedeno-Laurent J. Pan W.-C. Adamkiewicz G. Dominici F. (2014). Linking student performance in Massachusetts elementary schools with the “greenness” of school surroundings using remote sensing. *PLoS One* 9:e108548. 10.1371/journal.pone.0108548 25310542 PMC4195655

[B63] Zhu P. Zhang Y. (2008). Demand for urban forests in United States cities. *Landsc. Urban Plan.* 84 293–300. 10.1016/j.landurbplan.2007.09.005

[B64] Zuur A. F. Ieno E. N. Elphick C. S. (2009). A protocol for data exploration to avoid common statistical problems. *Methods Ecol. Evol.* 1 3–14. 10.1111/j.2041-210X.2009.00001.x

