# Supplementary material for: Might School Performance Grow on Trees? Examining the Link Between “Greenness” and Academic Achievement in Urban, High-Poverty Schools
Source: Front Psychol. 2018 Sep 25;9:1669. doi: 10.3389/fpsyg.2018.01669 (PMC6168033; doi:10.3389/fpsyg.2018.01669)
Supplement: Supplementary file 1 [file Data_Sheet_1.docx]

Supplementary Material

Can trees grow school performance? Examining the link between “greenness” and academic achievement in an urban, high-poverty school district

**Ming Kuo^a*^, Matthew H. E. M. Browning^b,d^, Sonya Sachdeva^c^, Kangjae Lee^d^, Lynne Westphal^c^**

*** Correspondence:** Corresponding Author: fekuo@illinois.edu

# Variance Inflation Factors for Table 4

In GLMs, variance inflation factors (VIFs) were below the recommended limit of 3.0, demonstrating multicollinearity did not bias results.

**Table 1.1**

VIFs for Table 4 in the main text

| **Predictors** | **VIF**^a^ |
| --- | --- |
| School trees | 1.31 |
| Neighborhood trees | 1.34 |
| %Disadvantaged | 1.72 |
| School trees*%Disadvantaged | 1.94 |
| Neighborhood trees*%Disadvantaged | 1.78 |

^a^values were the same for reading scores and math scores

**
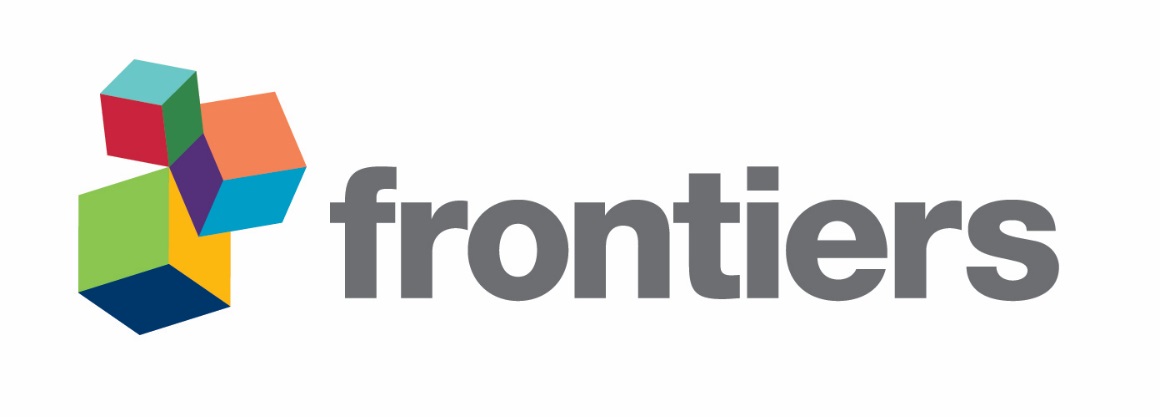
**
